# SARS-CoV-2 vaccine uptake, knowledge and attitude among health workers in Nairobi, Kenya: a quantitative study

**DOI:** 10.3389/fpubh.2026.1743484

**Published:** 2026-05-19

**Authors:** Olivia Nilsson, Gabriele Eiben, Sammy Wafula Simiyu, Kristina Carlén

**Affiliations:** 1 School of Health Sciences, University of Skövde, Skövde, Sweden; 2 Health Research Department, Nairobi City County, Nairobi, Kenya

**Keywords:** attitude, COVID-19, health workers, Kenya, knowledge, Nairobi, SARS-CoV-2, SARS-CoV-2 vaccine

## Abstract

**Introduction:**

The pandemic caused by SARS-CoV-2 is having, and will continue to have, devastating indirect consequences, particularly in developing countries. Understanding vaccine acceptance, knowledge, and attitudes among healthcare workers is crucial, as they influence public opinion, community uptake, and vaccination decision-making. This study aimed to identify variables associated with SARS-CoV-2 vaccine uptake, knowledge, and attitude among health workers in level 2 and 3 facilities in Nairobi, Kenya.

**Methods:**

A quantitative questionnaire was distributed to healthcare workers, and chi-square tests, Fisher’s exact tests, *t*-tests, and one-way ANOVA were conducted to determine associations between variables.

**Results:**

Vaccine uptake was significantly associated with knowledge index (*p* = 0.04), and nurses, assistant nurses, and physicians were more likely to be vaccinated than other health professions (*p* = 0.03). Knowledge index was significantly associated with age (*p* = 0.03), being female (*p* = 0.03), years of experience (*p* = 0.02), being vaccinated (*p* = 0.01), and attitude index (*p* < 0.01). A significant association was found between knowledge index and subcounty (*p* = 0.03). Dagoretti South, Embakasi East and Westlands had the lowest knowledge indexes among the subcounties. Attitude index was significantly associated with years of experience (*p* = 0.02), being vaccinated (*p* < 0.01), knowledge index (*p* < 0.01), and profession (*p* = 0.03).

**Discussion:**

Despite high vaccination uptake and generally positive attitudes, essential knowledge gaps regarding COVID-19 vaccines persist. The knowledge gaps include COVID-19 vaccine myths such as believing that, or being unsure whether, the vaccine can alter genes, increase autoimmune diseases, or cause COVID-19 disease to the receiver. These findings highlight the importance of addressing vaccine misconceptions and improving health literacy to strengthen vaccination strategies and pandemic preparedness among primary health workers.

## 1 Introduction

COVID-19 is a disease caused by the virus SARS-CoV-2 and is primarily affecting the respiratory tract. It was first detected in Wuhan, China, where the disease originated. The disease spread globally over a period of three to 4 months (1) and has been responsible for millions of deaths worldwide. Even though preventive measures have been able to limit morbidity and mortality, the introduction of new mutations of the virus and slackness in compliance with restrictions and recommendations have been shown to cause recurring infection waves, which sometimes can be more destructive than the first one (2). The pandemic caused by SARS-CoV-2 spread to nearly all countries worldwide and the rapid spread of COVID-19 globally led to a significant high death toll (3). Pandemics require collective global action to translate into global population immunity. Global population immunity can be accomplished by two pathways which are either through a previous infection or immunization. It is estimated by the World Health Organization that around two to 3 million deaths every year are prevented globally through immunization programs. Aiming for population immunity solely through a previous infection of COVID-19 could therefore lead to excess mortality and morbidity and a huge socioeconomic burden. Due to this, the ideal strategy to curb the catastrophic effects of COVID-19 is the initiation of mass immunization programs. However, this strategy is accompanied by two major hurdles: vaccine inequality and vaccine hesitancy, which leads to the necessity to have a two-pronged approach to attain global population immunity: promoting vaccine equality and combating vaccine hesitancy (2). Previous systematic reviews have demonstrated variability in COVID-19 vaccine acceptance among healthcare workers globally, particularly across low- and middle-income countries (4). Evidence from African healthcare settings even shows that although baseline knowledge is often acceptable, vaccination behaviour is still heavily shaped by safety concerns, misinformation, and institutional trust (5).

Kenya is one of the countries that was considered to have adopted moderate rather than strict measures in an attempt to balance the benefits and costs of the interventions. The Kenyan government introduced measures such as mandatory face masks in public, social distancing, a ban on large social gatherings indoors, and a requirement to present a negative COVID-19 test for arriving air passengers. The government also launched a COVID-19 vaccination campaign dubbed “Kenya tukae chonjo,” a Swahili slang phrase that means “we should stay vigilant.” The campaign aimed at increasing the rate of vaccination especially among the high-risk groups such as health workers, the older adult population, and people living with chronic conditions (6). The Kenyan healthcare system is divided into six levels of core health services, which are defined in four tiers of healthcare: community, primary care, county referral, and national referral. Level 1 equals the community care unit, characterized by the fact that no physical infrastructure is present. Level 2 (dispensaries and small clinics) and level 3 (health centers and small maternity clinics) constitute the primary healthcare service (7), which this study focused on.

Only 35% of the Kenyan adult population were fully vaccinated in September 2022, a number that 2 months later only had risen by 1.9% according to the Republic of Kenya Ministry of Health (8, 9). In Nairobi County, which has a relatively high literacy rate and an overall high educational level, the percentage of fully vaccinated people at about the same time was only 55.5% (9). This stands in sharp contrast to the results of a nationwide conducted study in Kenya in April 2020. This study showed that about 96% of the participants were willing to accept a COVID-19 vaccine and most of them were even willing to pay (10). According to a study conducted by Abdulle et al. (11), 71% of healthcare professionals in Kenya were willing to be vaccinated on condition that it would be freely available. However, it is not clear why almost 30% were not willing to be vaccinated.

The consequences of COVID-19 are, and have been, affecting global health, which gives rise to the importance of controlling the spread and minimizing the damage urgently (12). Since vaccination is the most essential public health measure and the most efficient strategy to keep the population safe from COVID-19, it was of great need to be prioritized at all levels. The necessity to vaccinate the general population as soon as possible during a disease outbreak is therefore a fact. A global survey showed that 48% of the population were confused about the COVID-19 vaccinations and were unsure whether they would get vaccinated or not (13). Another study found that only 54% of the participants intended to have the vaccine (14). The goal of obtaining herd immunity, which is the indirect protection from an infectious disease that occurs when a population is immune, requires a high level of vaccination acceptance in the population (14, 15). The vaccine hesitancy among the general population is directly associated with vaccine hesitancy among healthcare workers (16). Knowledge level has in addition repeatedly been identified as one of the strongest predictors of vaccination acceptance among healthcare workers (17). The vaccine acceptance, knowledge and attitude among healthcare workers are therefore important to understand and address since they directly influence public opinion, community vaccine acceptance, and decision-making regarding vaccination (18). The relationship between knowledge and attitudes in vaccination research is often described using Knowledge-Attitude-Practice (KAP) frameworks, where knowledge shapes attitudes that influence health behaviours, including vaccination uptake. Studies show that individuals with higher literacy and greater familiarity with vaccine information are more likely to accept vaccines, emphasizing the role of health literacy in shaping knowledge, attitudes, and practices (19). In other words, is information about healthcare workers’ COVID-19 vaccine uptake, knowledge and attitude crucial for policymakers and governments to determine possible reasons for the low vaccination numbers in the general population and to address the barriers to vaccine uptake (14). Studies that have been conducted on the uptake of COVID-19 vaccine among healthcare workers both worldwide and in Kenya specifically indicate a lower uptake than was expected based on official statistics (4, 11, 20). Exposure to vaccine misinformation has been shown to significantly reduce confidence in vaccine safety and vaccination intent (21, 22). A study carried out in Africa revealed a COVID-19 vaccine acceptance rate among healthcare workers in East Africa at 49% (16). It is furthermore necessary and utile to continuously follow up and gather information about variables such as vaccine uptake, knowledge level, and attitude regarding COVID-19 vaccination in the target group to facilitate the path towards herd immunity. As stated above, addressing possible hinderances might in turn have a butterfly effect on these areas at the community level. The possible myths and misconceptions about SARS-CoV-2 vaccine among healthcare workers ought to be revealed and addressed as soon as possible to boost vaccine uptake rates in Africa (16). Moreover, this investigation is supplementing current scientific information and guidelines regarding vaccines- and related health education. Despite previous studies on vaccine acceptance among healthcare workers in East Africa, there is limited evidence specifically examining SARS-CoV-2 vaccine uptake, knowledge, and attitudes of primary healthcare workers in level 2 and 3 facilities in Nairobi, which serve as critical points of care. To the research team’s knowledge, no such investigation has been conducted in Nairobi in this context. This study therefore provides crucial information to assess vaccine uptake, knowledge, and attitudes in this population, providing evidence that can inform targeted health education and vaccination strategies to strengthen pandemic preparedness.

## 2 Materials and methods

### 2.1 Study design and instruments

This research applied a quantitative study design, by using a questionnaire adapted from a similar study regarding knowledge and attitudes conducted in Bangladesh (14). The first section contained demographic data (see Table 1), and the second section was composed by 19 questions to which the participants could choose between the answers “agree,” “disagree” and “undecided/I do not know.” These questions resulted in a knowledge index and an attitude index that were each compared between different subgroups. The vaccination status was also compared between different subgroups to determine the significantly associated independent variables. The questionnaires were researcher administered, therefore any clarifications required by the respondents at the time of data collection were addressed. The administration time was 10–15 min.

**Table 1:** Demographics of the participants.

Characteristic	Category	*N*	%
Age	18–30	116	33.1
	31–40	112	32.0
	41–50	53	15.1
	50+	26	7.4
	Missing	43	12.3
Sex	Female	238	68.0
	Male	100	28.6
	Missing	12	3.4
Highest educational level	Primary	3	0.9
	Secondary	57	16.3
	Diploma	21	6.0
	Bachelor’s degree	204	58.3
	Master’s degree	8	2.3
	PhD	2	0.6
	Missing	55	15.7
Health profession	Care assistant	31	8.9
	Assistant nurse	5	1.4
	Nurse	108	30.9
	Physician	21	6.0
	Lab technologist	17	4.9
	Public health officer	7	2.0
	Other	144	41.1
	Missing	17	4.9
Workplace facility level	Level 2	69	19.7
	Level 3	281	80.3
	Missing	0	0.0
Years of professional experience	<3 years	91	26.0
	3–5 years	68	19.4
	6–9 years	56	16.0
	10+ years	95	27.1
	Missing	40	11.4
Total number of participants		350	100

Ethical approvals were obtained from National Commission for Science, Technology, and Innovation (NACOSTI) Ref No. NACOSTI/P/22/21812, the Daystar University Ethics Review Board, as well as official approvals from Nairobi County - Health Sector, each of the included sub-counties, and each concerned primary healthcare facility.

### 2.2 Participants

The study population was health professionals from level 2 and 3 healthcare facilities in Nairobi. They are classified as primary care units and therefore act as the first and principal point of care (23). Since the majority of the staff members in level 1 facilities are community volunteers and not educated healthcare workers they were not included. Eligible participants were health workers of 18 years and above. Participation was through voluntary basis. No incentives or monetary benefits were offered to participants who take part in this study. In level 2 and 3 healthcare facilities the most common health professions are nurses, physicians, assistant nurses, and public health technicians (24).

### 2.3 Power calculation

The sample size was aimed to be 384 participants; calculated by using Fisher’s formula with an estimated prevalence (P) of 50% as shown below. However, the target was almost met since the final number of participants was 350. The achieved sample size was considered adequate for exploratory association analyses; however, subgroup comparisons with small cell counts should be interpreted cautiously.


$$n=\frac{\left(Z^{2}*p*q\right)}{d^{2}}$$

*n* = sample size.

*z* = 95% confidence interval (usually 1.96).

*p* = prevalence number (50%) since it is known for the health care workers in level 2 and 3 health facilities in Nairobi.

*q* = (1−*p*).

*d* = maximum tolerable error, level of precision (0.05)


$$n=\frac{\left(1.96^{2}*0.50*0.50\right)}{0.05^{2}}$$

*n* = 384.16.

### 2.4 Sampling procedure

The data collection was carried out during the three first weeks of December 2022. The selection of health facilities was based on the advice of the subcounty Ministry of Health (MOH) of each subcounty, with the ambition to obtain a proportionate representation of health facilities by high-, middle- and low-income areas. Around 30–50% of the staff from each selected health facility was recruited due to availability and workload.

### 2.5 Statistical analysis

IBM SPSS Statistics 28 was the software used to conduct the statistical analyses. Analyses were conducted using available cases, and missing responses were not imputed. The prevalence of each vaccination status was calculated (“unvaccinated,” “vaccinated but not fully vaccinated” and “fully vaccinated”). Vaccination prevalence was calculated as the proportion of health workers in level 2 and 3 who were eligible for SARS-CoV-2 vaccine and had been vaccinated at the time of the study divided by the total number of participants. The knowledge index was based on 13 knowledge-related questions. Each correct answer added one point to the index score ranging from 0 to 13. Hence, the maximum index score per participant was 13. For descriptive purpose the index was divided into four levels, “very low” (0–4), “low” (5–7), “middle/high” (8–10) and “very high” (11–13). For the bivariate analysis, the variables were divided into two group: age: >35 and ≤35 years, gender: male and female, subcounty: lower/middle income and higher income, educational level: <bachelors degree and ≥ bachelors degree, health profession: nurses and physicians, and others, facility level: level 2 and level 3, years of experience: >5 and ≤5 years, knowledge index: lower (0–7) and higher (8–13), and attitude index: lower (0–6) and higher (7–12). The attitude index for COVID-19 vaccines was calculated by coding the positive attitude answer as 2, undecided as 1, and the negative attitude answer as 0. Since there were six attitude related questions the maximum attitude index score was 12. This index analysis was used by Islam et al. (14) to investigate COVID-19 vaccine knowledge and attitudes in Bangladesh. Cut-off values for age, experience, knowledge index and attitude index were defined *a priori* to facilitate interpretation and comparability with similar knowledge-attitude-practice studies.

The three dependent variables vaccination status, knowledge index, and attitude index were analysed separately to determine which independent variables were significantly associated with each of them. Chi square tests, Fisher’s exact tests, *t*-tests, and one-way ANOVA tests were used. The study aimed to explore potential associations rather than identify independent predictors; therefore, bivariate analyses were used as an exploratory analytical approach. A *p*-value of < 0.05 was considered statistically significant. Possible interferences include the cultural aspect regarding the construction of the questionnaire which may influence the variables and the analyses. Therefore, local contact and collaboration points were consulted in each step of the process.

## 3 Results

### 3.1 Demographics

The most common age groups were 18–30 (33.1%) and 31–40 years (32.0%). The participants mean age was 34.5 (SD = 9.26) and the age ranged from 19 to 58 years. There was an over representation of females (68.0%) in the study. The most common educational level among the participants was a bachelor’s degree (58.3%) and the most common specified profession was nurse (30.9%). There was an over representation of participants working in a level 3 health facility (80.3%), compared to the participants working in a level 2 health facility (19.7%). The most common number of years of professional experience was 10+ and <3 years of professional experience (27.1 and 26.0% respectively). For further detailed demographic information see Table 1.

### 3.2 Descriptive statistics

#### 3.2.1 Vaccination status

The proportion of the participants that were fully vaccinated (2 doses) were 85.4% (Table 2). The vaccinated but not fully vaccinated were 7.7% of the participants, and 2.0% were unvaccinated voluntarily. No participants were unvaccinated due to vaccine availability. However, 4.9% of the answers to the vaccine status question were missing.

**Table 2:** The prevalence of SARS-CoV-2 vaccination status amongst the participants.

Vaccination status	*N*	%
Fully vaccinated (2 doses)	299	85.4
Vaccinated but not fully vaccinated (1 dose)	27	7.7
Unvaccinated (voluntarily)	7	2.0
Unvaccinated (vaccine availability)	0	0.0
Missing	17	4.9
Total	350	100.0

#### 3.2.2 Knowledge index

The most common knowledge index (KI) level (53.1%) was level *middle/high* (Table 3) which accounts for roughly 60–75% correct answers. The second most common knowledge index (23%) was level *very high* which accounts for around >85% correct answers. The overall mean knowledge index was 8.9 (SD = 2.01). In this study, the subcounties with the highest knowledge index regarding SARS-CoV-2 vaccines were Roysambu (9.5) and Dagoretti North (9.5), while Embakasi East (8.2) and Dagoretti South (8.1) were the ones with the lowest found knowledge indexes. For further information regarding the subcounty KIs please see Supplementary Table 1.

**Table 3:** Knowledge levels of the participants.

Knowledge index level	*N*	%
Very low	12	3.4
Low	69	19.7
Middle/high	186	53.1
Very high	83	23.7
Total	350	100.0

#### 3.2.3 Attitude index

The attitude index was based on 6 attitude-related questions (see Supplementary Table 2, questions 14–19). On a scale from 0 to 12, the most common attitude index was 12. The overall mean attitude index was 10.6 (SD = 2.14) which accounts for 88% of the maximum possible attitude score.

### 3.3 Inferential statistics

#### 3.3.1 Vaccine status: association with independent variables

The Chi-Square tests of independence and Fisher’s exact tests determined a significant association between vaccination status and the variables: health profession, *χ*^2^ (1, *N* = 327, *p* = 0.03), and knowledge index level, *p* = 0.04, which was analysed using Fisher’s exact test since the *χ*^2^ results were invalid. The participants with higher knowledge index were more likely to be vaccinated than the participants with lower knowledge index. No other significant relationship was determined (Tables 4 and 5).

**Table 4:** *χ*^2^ tests to determine association with vaccination status.

Dependent variables	Independent variables	Category	% Unvaccinated within category	*N*	*χ* ^2^	*p*-value
Vaccination status *Unvaccinated* *Vaccinated*	Age	≤35	1.1	187	3.33	0.07
		>35	4.3	116		
	Gender	Female	1.3	234	2.73	0.10
		Male	4.2	96		
	Subcounty	Lower/middle income	1.9	215	0.17	0.68
		Higher income	2.5	118		
	Health profession	Group 1 *(Nurse, As. Nurse, Physician)*	0.0	133	4.90	***0.03***
		Group 2 *(HIV Testing Services, Lab, Public Health Officers, others)*	3.6	194		
	Years of experience	≤5 years	0.6	157	1.99	0.16
		>5 years	2.7	149		
		Higher	1.6	312		

Bold italic *p*-values indicate statistically significant associations (*p* < 0.05).

**Table 5:** Fisher’s exact test to determine association with vaccination status (applied on variables with invalid *χ*^2^-results).

Variables	Category	% Unvaccinated within category	*N*	*p*-value
Educational level	<Bachelors	2.6	77	0.61
	≥Bachelors	1.4	210	
Facility level	Level 2	4.6	65	0.14
	Level 3	1.5	268	
Knowledge index level	Lower	7.5	40	***0.04***
	Higher	1.4	293	
Attitude index level	Lower	9.5	21	0.07
	Higher	1.6	312	

Bold italic *p*-values indicate statistically significant associations (*p* < 0.05).

#### 3.3.2 Knowledge: association with independent variables

The independent samples *t*-tests determined that the knowledge index was significantly higher among participants older than 35 years (*M* = 9.26, SD = 1.90) than participants younger than or 35 years (*M* = 8.78, SD = 1.89), *p* = 0.03 (Table 6). The knowledge index was significantly higher among females (*M* = 9.10, SD = 1.92) than males (*M* = 8.58, SD = 2.14), *p* = 0.03. The independent samples *t*-test also determined that the knowledge index was significantly higher among the participants with more experience (*M* = 9.26, SD = 1.93) than the ones with less experience (*M* = 8.75, SD = 1.88), *p* = 0.02. It also determined that the knowledge index was significantly higher among the vaccinated (*M* = 9.02, SD = 1.93) than the unvaccinated (*M* = 7.00, SD = 3.26), *p* = 0.01. The independent samples *t*-test determined in addition a significantly higher knowledge index among the group with higher attitude index level (*M* = 9.13, SD = 1.78) than the group with lower attitude index level (*M* = 6.00, SD = 2.80), *p* ≤ 0.01.

**Table 6:** Independent samples *t*-tests performed with knowledge index as dependent variable.

Dependent variable	Independent variable	Categories	*N*	Mean	Std.	*T*	*p*-value
Knowledge index (KI)	Age	Younger	189	8.78	1.89	−2.16	***0.03***
		Older	118	9.26	1.90		
	Gender	Female	238	9.10	1.92	2.19	***0.03***
		Male	100	8.58	2.14		
	Educational level	Lower	81	9.11	1.85	1.27	0.20
		Higher	214	8.77	2.12		
	Facility level	Level 2	69	8.54	2.13	−1.82	0.07
		Level 3	281	9.02	1.97		
	Years of experience	≤5 years	159	8.75	1.88	−2.35	***0.02***
		>5 years	151	9.26	1.93		
	Vaccination status	Unvaccinated	7	7.00	3.26	−2.70	***0.01***
		Vaccinated	326	9.02	1.93		
	Attitude index level	Lower	22	6.00	2.80	−7.63	***<0.01***
		Higher	328	9.13	1.78		

Bold italic *p*-values indicate statistically significant associations (*p* < 0.05).

The one-way ANOVA tests revealed that there was a statistically significant difference in knowledge index between at least two subcounty groups (*F*(10, 339) = 2,00, *p* = 0.032). The one-way ANOVA test determined no statistically significant difference in knowledge index within the health profession groups (*F*(6, 326) = 1,54, *p* = 0.165).

#### 3.3.3 Attitude: association with independent variables

Independent samples *t*-tests revealed that the attitude index was significantly higher among the healthcare workers with more experience (*M* = 10.9, SD = 1.64) than the ones with less experience (*M* = 10.4, SD = 2.17), *p* = 0.02. It also determined that the attitude index was significantly higher among the vaccinated (*M* = 10.6, SD = 2.11) than the unvaccinated (*M* = 7.9, SD = 2.55), *p* ≤ 0.01. The independent samples *t*-tests determined in addition significantly higher attitude index among the group of higher knowledge index level (*M* = 10.9, SD = 1.79) than the group with lower knowledge index level (*M* = 8.5, SD = 2.97), *p* ≤ 0.01 (Table 7).

**Table 7:** Independent samples *t*-tests performed with attitude index as dependent variable.

Dependent variable	Independent variable	Categories	*N*	Mean	Std.	*T*	*p*-value
Attitude index (AI)	Age	Younger	189	10.39	2.31	−1.68	0.10
		Older	118	10.81	1.93		
	Gender	Female	238	10.66	1.89	1.15	0.25
		Male	100	10.37	2.65		
	Educational level	Lower	81	10.51	2.15	−0.11	0.10
		Higher	214	10.51	2.26		
	Facility level	Level 2	69	10.39	1.93	−0.74	0.46
		Level 3	281	10.60	2.19		
	Years of experience	≤5 years	159	10.43	2.17	−2.31	***0.02***
		>5 years	151	10.93	1.64		
	Vaccination status	Unvaccinated	7	7.86	2.55	3.44	***<0.01***
		Vaccinated	326	10.63	2.11		
	Knowledge index level	Lower	45	8.47	2.97	−7.60	***<0.01***
		Higher	328	10.87	1.79		

Bold italic *p*-values indicate statistically significant associations (*p* < 0.05).

The One-way ANOVA tests revealed that there was a statistically significant difference in attitude index between at least two health profession groups (*F*(6, 326) = 2.44, *p* = 0.03). The one-way ANOVA test determined no statistically significant difference in attitude index within the subcounty groups (*F*(10, 339) = 1.63, *p* = 0.10).

## 4 Discussion

This study revealed a fully vaccinated prevalence of 85% while determining the variables knowledge index level as positively associated with COVID-19 vaccination status. Health profession was in addition found to be a variable significantly associated with vaccination status. The participants were more likely to be vaccinated if they were nurses, assistant nurses or physicians compared to other professions such as lab technicians, HIV testing services and public health officers. Being >35 years old, female, vaccinated, having >5 years of professional experience and a higher attitude index level were found positively significantly associated with knowledge index level. In addition, there was a statistically significant difference in knowledge index between at least two of the subcounty groups. Having >5 years of experience, being vaccinated and having a higher knowledge index level were found to be positively significantly associated with attitude index level. In addition, there was a statistically significant difference in attitude index between at least two of the health professions.

### 4.1 Vaccine uptake

A systematic review and meta-analysis focused on the general public in East Africa conducted by Alemayehu et al. revealed that the variables knowledge, attitude, gender, and history of COVID-19 infection have a significant association with COVID-19 vaccine acceptance (25). Even though vaccine acceptance and vaccine uptake do not per definition have the same meaning, they often walk hand in hand, and in this study, knowledge was found to be significantly associated with vaccine uptake, which further strengthens the results of the study conducted by Alemayehu. However, the results differ regarding attitude and gender. This leaves one to wonder whether the imbalance of males and females could have had an impact on the results since there were almost 2.5 times more females than males participating in this study. With a larger sample the results regarding this variable might have been different. The variable history of COVID-19 infection was not included in this research project.

Adane et al. investigated knowledge, attitude and perception about COVID-19 vaccines among healthcare workers in Ethiopia and also found the variables knowledge and attitude to be significantly related to the willingness to get vaccinated (26). Adane et al. did not find a significant relationship between willingness to get vaccinated and health profession (26). However, Farah et al. found significant differences between health profession categories when they investigated disparities in COVID-19 vaccine uptake among healthcare workers (27). Health profession was also determined a significantly associated variable with vaccine uptake in this study.

Since knowledge index was found to be positively significantly associated with SARS-CoV-2 vaccination status in this study, it was essential for the authors to highlight the vaccination coverage in the general population in Nairobi and Kenya during the same time period. However, the links from where this particular information was gathered, the daily COVID-19 reports from the Republic of Kenya MOH (8, 9), are as of writing this article not working.

### 4.2 Knowledge

The results highlight the current knowledge gaps among the healthcare workers in level 2 and level 3 health facilities in Nairobi County and are particularly of interest since knowledge was found to be significantly associated with vaccine uptake. The knowledge gaps were specifically highlighted by the fact that 24.2% of the participants answered either “Agree” or “I do not know” to “COVID-19 vaccines can alter my genes,” that 74% answered either “Agree” or “I do not know” to the statement “COVID-19 vaccines contain live viruses,” that 19.9% answered either “Agree” or “I do not know” to “COVID-19 vaccines can make me sick with COVID-19” and that 30.8% answered either “Agree” or “I do not know” to “Vaccination against COVID-19 does increase autoimmune diseases.” These findings are consistent with broader evidence showing that beliefs in COVID-19 misinformation, including inaccurate assumptions about vaccine composition and effects, have been documented in various settings (21, 22). Possible health education interventions should be focused on the physical properties, the effectiveness and the side effects of the COVID-19 vaccines to increase health promotion and health literacy.

Misconceptions regarding vaccine composition and biological effects have been reported internationally, including among healthcare workers (22). Comparing with the results from Islam et al. (14) the statements that were similar in both studies generated results around the same percentages. The overall correct answers rate in the study by Islam et al. (14) was 57% compared to the 68.7% in this study. Again, this might be explained by the difference in time or by country and/or continent specific differences. A study conducted by Adane et al. in the bordering country Ethiopia investigated knowledge, attitude, and perceptions of COVID-19 vaccines among healthcare workers (26). Among the 404 participants the mean knowledge score was 68.0% which is similar to the 68.7% that represents the mean knowledge score of the healthcare workers in this study. This information might further validate the results of both East African studies.

A study conducted by Juin et al. investigated knowledge, attitude, and practices of COVID-19 vaccination among adults in Singapore, and also determined that the variables vaccination status and attitude score were significantly associated with knowledge score (28). When taking a closer look at the different subcounty means, Embakasi South, Westlands, Embakasi East and Dagoretti South all stand out since they have a mean knowledge score of below the general mean value. The highest mean knowledge indexes were found in Roysambu (*M* = 9.5, SD = 1.52) and Dagoretti North (*M* = 9.5, SD = 2.18).

Elhadi et al. (29) conducted a cross-sectional study in Libya to assess knowledge, attitudes, and acceptance of the COVID-19 vaccine among both healthcare workers and the general population, including medical students, physicians, and paramedics. Data were collected through an online questionnaire distributed via digital platforms. This is a method that likely facilitated a diverse respondent pool but may also have introduced selection bias and influenced response accuracy due to the self-administered format.

In contrast, the present study provides important insights into SARS-CoV-2 vaccine uptake, knowledge, and attitudes within a specific professional group – primary healthcare workers in level 2 and level 3 health facilities in Nairobi. Using a paper-based questionnaire allowed for direct engagement with participants and potentially more controlled data collection. This design aimed to explore how levels of knowledge and attitudes among primary healthcare workers relate to actual vaccination uptake, with a particular focus on the link between higher knowledge and increased willingness to be vaccinated.

While both studies assessed similar constructs, they differed in target populations, regional contexts, data collection approaches, and resulting conclusions. Elhadi et al. (29) reported generally high levels of knowledge and positive attitudes towards COVID-19 vaccination, with no statistically significant difference between healthcare workers and the general population. Conversely, our findings indicate that although attitudes towards SARS-CoV-2 vaccination among primary healthcare workers in Nairobi were largely positive, notable knowledge gaps remain. The gaps are particularly concentrated around vaccine composition and mechanisms, which could influence future vaccine promotion strategies.

### 4.3 Attitude

The mean attitude index score in this study was 10.56 (SD = 2.14) which accounts for 88% of the maximum attitude index value. Adane et al. (26) found an average attitude score percentage of 73.9% and Islam et al. (14) found an average attitude index percentage at 77.8%. Both these results are also in the higher percentages although they differ a bit from the results of this study. Possible explanations to these differences could be the difference in time in regard to the study dates or country specific differences. Another possible explanation could be social desirability bias, in which healthcare workers answered questions in a manner that would be viewed favourably by others which may have resulted in over-reporting of good attitudes.

Neither Adane et al. (26) nor Islam et al. (14) investigated independent variables associated with attitude regarding COVID-19 vaccine. However, a study conducted by Tolossa et al. investigated this among health professionals in Ethiopia and revealed a significant association between attitude and the variable knowledge, which was also determined in this study (30). Tolossa et al. did not investigate the variable years of experience (30). However, a study by Demir et al. also revealed that the variable years of professional experience had a significant impact on health workers attitude. It should be highlighted that the attitude towards a specific SARS-CoV-2 vaccine was investigated in the study by Demir el al. and not attitude towards SARS-CoV-2 vaccines in general (31). The mentioned studies strengthen the results of this research since this study also revealed that knowledge and years of experience were significantly associated with attitude index.

### 4.4 Limitations

This study has several limitations. As a cross-sectional exploratory study, causal relationships cannot be established, and some confounding between demographic factors (e.g., profession, experience, and gender) may remain. Multivariable regression analyses were not performed, so the associations reported are unadjusted. Although the knowledge and attitude indices were constructed based on prior studies and expert input, their internal consistency was not formally validated (e.g., Cronbach’s alpha). This may limit the reliability of the indices.

The time limitation was the reason for not meeting the participant number target of 384. However, it was almost reached since 350 health workers participated in this study. As stated above, several studies that were conducted in Kenya, East Africa or other countries revealed similar results, which validate the results of this study. The 350 participants may therefore be enough to draw preliminary conclusions about the population.

The original plan was to through a randomization and stratification process choose the data collection facilities. Because of the delays and time restriction this was not possible. In each subcounty MOH meeting the principal investigator asked for approval to collect data from four to six facilities within the subcounty. In addition, it was communicated to the MOH that the largest level 2 and level 3 facilities were preferred, which was done to facilitate meeting the target number of participants withing the short amount of time. The fact that the data collection sites were not randomized could have affected the results. Researcher-administered questionnaires may have in addition resulted in social desirability bias, particularly in attitude-related questions.

As the data was processed it became evident that the questionnaire choices for health profession were insufficient despite the fact that regional experts within local health systems were consulted in various steps of the questionnaire construction process. A total of 41% marked the “Other” option, and a majority of those did not write their specific profession where indicated. Out of those who marked “Other” and specified their answer, many were HIV-testing services counsellors and clinicians. This may have affected the results involving health profession as an independent variable. Another important limitation is that some professional subgroups were small, which may reduce the stability of subgroup comparisons. In addition, missing data for some variables may have introduced bias.

To obtain valid test results of the independent variables educational level, facility level, knowledge index, and attitude index when analysing vaccine uptake as dependent variable, Fisher’s exact test was used. Out of mentioned variables, knowledge index level was determined to be significantly associated with vaccination status. Surprisingly, the attitude level was not determined to be significantly associated with vaccination status. This was unexpected since 9.5% of the lower attitude index group was unvaccinated while only 1.6% were unvaccinated in the higher attitude index group. Reasons for that outcome could be the group sizes or the sample size, since the result of the statistical analysis is dependent on these factors. Other studies determined attitude level as significantly associated with vaccination status (25, 26).

## 5 Conclusion

This study revealed major knowledge gaps but in general a positive attitude towards SARS-CoV-2 vaccinations and a high vaccine uptake among healthcare workers in level 2 and level 3 health facilities in Nairobi. Knowledge was found to be positive significantly associated with vaccination status, and that in combination with the fact that healthcare personnel influence the general population, suggests that health education programs, vaccine promoting interventions and more accurate information should be distributed to healthcare personnel. Based on the results from this study, possible health education interventions should be focused on the physical properties, the effectiveness and the side effects of the COVID-19 vaccines to increase health promotion and health literacy. The results emphasize the key role of healthcare workers’ health literacy in shaping their knowledge, attitudes, and practices (KAP) regarding SARS-CoV-2 vaccination. Improving health literacy may reduce misconceptions, support informed vaccine decisions, and increase uptake in the communities they serve.

This information might provide additional guidance regarding the health literacy aspect in pandemic action plans or know-hows, as well as knowledge on how to further on increase vaccination coverage through primary health workers. Since healthcare personnel have a major influence among the general population these targeted interventions would increase knowledge and vaccine rates substantially both among healthcare personnel within level 2 and level 3 facilities, but also among the general population. Lastly, this study also lays a foundation for future research on SARS-CoV-2 vaccination, supporting a better understanding of healthcare professionals’ knowledge and practices in level 2 and level 3 facilities in Nairobi, and potentially enhancing population-level vaccine coverage. Future studies could follow changes in knowledge and attitudes over time, especially with booster campaigns and new vaccines. Larger, randomized studies could examine subgroup differences more rigorously and control for confounders, while qualitative research could explore the persistent misconceptions and barriers influencing healthcare workers’ vaccination practices.

## Data Availability

The original contributions presented in the study are included in the article/Supplementary material, further inquiries can be directed to the corresponding author.
