# Supplementary material for: SARS-CoV-2 vaccine uptake, knowledge and attitude among health workers in Nairobi, Kenya: a quantitative study
Source: Front Public Health. 2026 May 19;14:1743484. doi: 10.3389/fpubh.2026.1743484 (PMC13226465; doi:10.3389/fpubh.2026.1743484)
Supplement: Supplementary file 1 [file Table_1.DOCX]

Supplement 1

*Supplement table 1. The knowledge index means of each subcounty.*

| **Subcounty** | **N** | **Mean** | **SD** |
| --- | --- | --- | --- |
| Roysambu | 46 | 9.5 | 1.52 |
| Dagoretti North | 22 | 9.5 | 2.18 |
| Ruaraka | 38 | 9.3 | 1.58 |
| Embakasi West | 3 | 9.3 | 1.53 |
| Makadara | 30 | 9.2 | 1.67 |
| Langata | 24 | 9.1 | 1.93 |
| Starehe | 44 | 9.1 | 1.74 |
| Embakasi South | 29 | 8.9 | 1.90 |
| Westlands | 35 | 8.6 | 2.37 |
| Embakasi East | 54 | 8.2 | 2.04 |
| Dagoretti South | 25 | 8.1 | 3.01 |
