# Supplementary material for: SARS-CoV-2 vaccine uptake, knowledge and attitude among health workers in Nairobi, Kenya: a quantitative study
Source: Front Public Health. 2026 May 19;14:1743484. doi: 10.3389/fpubh.2026.1743484 (PMC13226465; doi:10.3389/fpubh.2026.1743484)
Supplement: Supplementary file 2 [file Table_2.DOCX]

Supplement 2

| *Supplement table 2. Distribution of the participants' answers to the questionnaire statements* | | | | |
| --- | --- | --- | --- | --- |
| **Statement from questionnaire** | **Answers** | **% within statement*** | **% unvaccinated within answer**** | **% vaccinated within answer***** |
| 1. COVID-19 is a great threat to global health | Agree | 94.5 | 2.2 | 97.8 |
|  | Disagree | 2.9 | 0.0 | 100 |
|  | I don’t know | 2.6 | 0.0 | 100 |
| 2. Covid-19 is not real | Agree | 6.6 | 4.3 | 95.7 |
|  | Disagree | 87.6 | 1.7 | 98.3 |
|  | I don’t know | 5.7 | 5.6 | 94.4 |
| 3. I know about COVID-19 vaccines | Agree | 97.4 | 2.2 | 97.8 |
|  | Disagree | 0.9 | 0.0 | 100 |
|  | I don’t know | 1.8 | 0.0 | 100 |
| 4. I know about the effectiveness of COVID-19 vaccines | Agree | 92.5 | 1.3 | 98.7 |
|  | Disagree | 2.0 | 0.0 | 100 |
|  | I don’t know | 5.5 | 17.6 | 82.4 |
| 5. COVID-19 vaccines contain live viruses | Agree | 58.9 | 1.1 | 98.9 |
|  | Disagree | 26.0 | 1.2 | 98.8 |
|  | I don’t know | 15.1 | 8.3 | 91.7 |
| 6. It is dangerous to overdose COVID-19 vaccines | Agree | 72.1 | 2.5 | 97.5 |
|  | Disagree | 8.1 | 0.0 | 100 |
|  | I don’t know | 19.8 | 1.6 | 98.4 |
| 7. Vaccines against COVID-19 can prevent serious illness and death caused by COVID-19 | Agree | 91.0 | 1.7 | 98.3 |
|  | Disagree | 6.1 | 5.0 | 95.0 |
|  | I don’t know | 2.9 | 10.0 | 90.0 |
| 8. It is not possible to reduce the incidence of COVID-19 without vaccination | Agree | 54.0 | 1.1 | 98.9 |
|  | Disagree | 42.8 | 2.1 | 97.9 |
|  | I don’t know | 3.2 | 20.0 | 80.0 |
| 9. Vaccination against COVID-19 does increase allergic reactions | Agree | 16.7 | 1.8 | 98.2 |
|  | Disagree | 70.7 | 1.3 | 98.7 |
|  | I don’t know | 12.6 | 8.1 | 91.9 |
| 10. COVID-19 can alter my genes | Agree | 5.2 | 0.0 | 100 |
|  | Disagree | 75.9 | 1.6 | 98.4 |
|  | I don’t know | 19.0 | 5.1 | 94.9 |
| 11. COVID-19 vaccines can make me sick with COVID-19 | Agree | 11.1 | 5.3 | 94.7 |
|  | Disagree | 80.1 | 0.8 | 99.2 |
|  | I don’t know | 8.8 | 115 | 88.5 |
| 12. Vaccination against COVID-19 does increase autoimmune diseases | Agree | 15.7 | 2.0 | 98.0 |
|  | Disagree | 59.2 | 1.6 | 98.4 |
|  | I don’t know | 25.1 | 3.8 | 96.2 |
| 13. Side effects of vaccines against COVID-19 are mostly mild | Agree | 81.0 | 1.9 | 98.1 |
|  | Disagree | 13.7 | 0.0 | 100 |
|  | I don’t know | 5.2 | 13.3 | 86.7 |
| 14. I am afraid of the side effects of vaccines against COVID-19 | Agree | 28,1 | 5,6 | 94,4 |
|  | Disagree | 64,6 | 0,5 | 99,5 |
|  | Undecided | 7,2 | 4,3 | 95,7 |
| 15. Everybody should get vaccinated against COVID-19 | Agree | 87,6 | 1,0 | 99,0 |
|  | Disagree | 8,4 | 10,3 | 89,7 |
|  | Undecided | 4,0 | 8,3 | 91,7 |
| 16. I trust in government information regarding COVID-19 | Agree | 88,8 | 2,0 | 98,0 |
|  | Disagree | 4,0 | 0,0 | 100,0 |
|  | Undecided | 7,2 | 4,3 | 95,7 |
| 17. I encourage my friends and family to get vaccinated against COVID-19 | Agree | 94,3 | 1,6 | 98,4 |
|  | Disagree | 2,3 | 12,5 | 87,5 |
|  | Undecided | 3,4 | 9,1 | 90,9 |
| 18. I trust in the efficiency of COVID-19 vaccines | Agree | 88,2 | 1,4 | 98,6 |
|  | Disagree | 3,8 | 7,7 | 92,3 |
|  | Undecided | 8,1 | 7,4 | 92,6 |
| 19. COVID-19 vaccines are safe | Agree | 88,5 | 1,4 | 98,6 |
|  | Disagree | 2,0 | 0,0 | 100,0 |
|  | Undecided | 9,5 | 10,0 | 90,0 |
| ** Distribution of answers within statement*  *** Percentage of participants within the specific answer who were unvaccinated*  **** Percentage of participants within the specific answer who were vaccinated* | | | | |
